# *Hypericum perforatum *treatment: effect on behaviour and neurogenesis in a chronic stress model in mice

**DOI:** 10.1186/1472-6882-11-7

**Published:** 2011-01-27

**Authors:** Rosalia Crupi, Emanuela Mazzon, Angela Marino, Giuseppina La Spada, Placido Bramanti, Fortunato Battaglia, Salvatore Cuzzocrea, Edoardo Spina

**Affiliations:** 1 Department of Clinical and Experimental Medicine and Pharmacology, School of Medicine, University of Messina, via Consolare Valeria, 98125 Messina, Italy; 2 IRCCS Centro Neurolesi "Bonino-Pulejo", via Provinciale Palermo, C. da Casazza 98124 Messina, Italy; 3 Department of Life Sciences "M.Malpighi", Section of General Physiology and Pharmacology, University of Messina, via F. Stagno D'Alcontres 31, 98166 Messina, Italy; 4 Department of Psychiatry, Columbia University, New York College of Podiatric Medicine, 53 East 124thStreet, New York, 10035 NY, USA

## Abstract

**Background:**

Extracts of *Hypericum perforatum *(St. John's wort) have been traditionally recommended for a wide range of medical conditions, in particular mild-to-moderate depression. The present study was designed to investigate the effect of Hypericum perforatum treatment in a mouse model of anxiety/depressive-like behavior, induced by chronic corticosterone administration.

**Methods:**

CD1 mice were submitted to 7 weeks corticosterone administration and then behavioral tests as Open Field (OF), Novelty-Suppressed Feeding (NSF), Forced Swim Test (FST) were performed. Cell proliferation in hippocampal dentate gyrus (DG) was investigated by both 5-bromo-2'-deoxyuridine (BrdU) and doublecortin (DCX) immunohistochemistry techniques and stereological procedure was used to quantify labeled cells. Golgi-impregnation method was used to evaluate changes in dendritic spines in DG. Hypericum perforatum (30 mg/Kg) has been administered for 3 weeks and then neural development in the adult hippocampus and behavioral changes have been examined.

**Results:**

The anxiety/depressive-like state due to chronic corticosterone treatment was reversed by exogenous administration of Hypericum perforatum; the proliferation of progenitor cells in mice hippocampus was significantly reduced under chronic corticosterone treatment, whereas a long term treatment with Hypericum perforatum prevented the corticosterone-induced decrease in hippocampal cell proliferation. Corticosterone-treated mice exhibited a reduced spine density that was ameliorated by Hypericum perforatum administration.

**Conclusion:**

These results provide evidence of morphological adaptations occurring in mature hippocampal neurons that might underlie resilient responses to chronic stress and contribute to the therapeutic effects of chronic Hypericum perforatum treatment.

## Background

Extracts of *Hypericum perforatum *(St. John's wort) have been traditionally recommended for a wide range of medical conditions [1]. The most common modern-day use of St. John's wort is the treatment of depression [2]. In this respect, different controlled trials have confirmed its efficacy in the treatment of mild-to-moderate depression [3]. In addition, several studies indicated that *Hypericum perforatum *demonstrated anxiolytic and anti-inflammatory effects [4,5]. *Hypericum *extract contains numerous compounds with documented biological activity such as the naphthodianthrones hypericin and pseudohypericin, a broad range of flavonoids including hyperoside, isoquercitrin and quercetin, and the phloroglucinols hyperforin and pseudohyperforin [6]. With regard to this, Muller et al. [7] have demonstrated that *Hypericum perforatum *inhibits the reuptake of several synaptosomal neurotransmitters such as serotonin, noradrenaline, dopamine, with efficiencies similar to that affinity selective inhibitors [8] and modulates neuronal excitability via glutamatergic and GABAergic mechanisms [4]. Nevertheless, the function of *Hypericum perforatum *in the mammalian brain and its role in modulating affective behaviours remain still unclear. For instance, basic, behavioral, and clinical evidences indicate various neuroendocrine, autonomic, and behavioral adaptative responses (resilience) to acute and chronic stress that are critical to the occurrence of anxious and depressive illnesses [9,10]. Moreover, changes in hippocampal plasticity are important biomarkers of stress response and adaptation and play a remarkable role in both pathogenesis of mood disorders and mechanisms of action of antidepressants [11,12]. Stress causes alterations in hippocampal networks, including altered patterns of neurogenesis and remodeling of dendrites [13,14]. In the adult brain, progenitor cells in the subgranular zone (SGZ) can give rise to newborn neurons which can migrate into the granule cell layer where they differentiate into granular neurons and are then functionally integrated into the hippocampal circuitry [15,16]. Chronic stress and direct corticosterone administration in animal models, decreases adult hippocampal neurogenesis [17-20] and increased apoptosis of newly generated neurons in the hippocampus [21]. In addition, chronic stress and *in-vivo *corticosterone administration induced dendritic morphological changes in the CA3 region of the hippocampus [20-24]. There is growing evidence that many of these chronic stress-induced structural changes in the hippocampus can be reversed by antidepressant treatment [27-29]. Hence, in this study we aimed to further investigate the antidepressant-like and antianxiety-like activity of *Hypericum perforatum *extract using a chronic stress model in mice. In particular we attempted to ascertain whether *Hypericum perforatum *might revert the effects of corticosterone administration on hippocampal progenitor cell proliferation and dendritic spine density and explored the behavioral correlations.

## Methods

### Animals

Adult male CD1 mice (Harlan Nossan, Milan, Italy) were housed four to five per cage and in a 12 h (7:00 AM to 7:00 PM) light-dark cycle at 21 ± 1°C and 50 ± 5% humidity with food and water available *ad libitum*. All corticosterone - treated mice were 7-8 weeks old and weighed 25-36 g at the beginning of the treatment. Mice were acclimated to the behavioral room at for 2 hours prior the testing. Behavioral tests were administered during the light phase and were conducted in compliance with the NIH (National Institutes of Health) laboratory animal care guidelines. The animal protocol was approved by the Institutional Animal Care and Use Committee (Council directive # 87-848, October 19, 1987, Ministère de l'Agriculture et de la Forêt, Service Vétérinaire de la Santé et de la Protection Animale, permission # 92-256 to D.J.D.). Animal care was in compliance with Italian regulations on protection of animals used for experimental and other scientific purposes (D.M. 116192) as well as with the EEC regulations (O.J: of E.C.L 358/1 12/18/1986).

### Drugs and treatment schedule

Corticosterone and BrdU (5-Bromo-2'-deoxyuridine) were purchased from Sigma Aldrich (Milan, Italy). *Hypericum perforatum *methanolic extract was a kind gift of Indena (Milan, Italy) and it was defined by the producer as containing 0.34% of hypericin, 4.1% of hyperforin, 5% of flavonoids (rutin, kaempferal, luteolin, myricetin, quercetin, quercitrin, and isoquercitrin), 10% tannins, and the remaining by maltodextrins.

**Figure 1:**
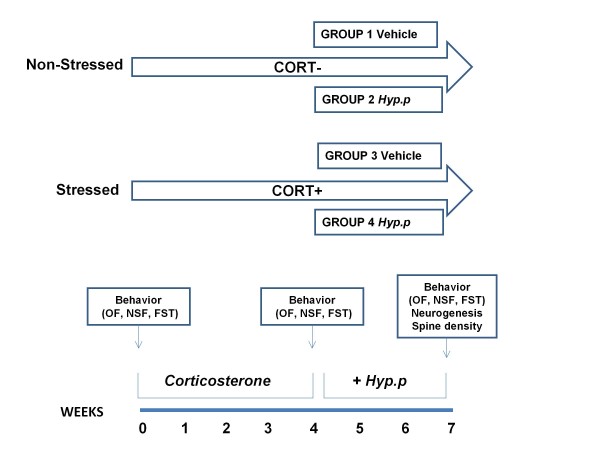
**Experimental design**. CD1 mice were administered during 7 weeks with vehicle or corticosterone in the presence or absence of *Hypericum perforatum *during the last three weeks of the corticosterone regimen. At the end of treatment the animals were tested in the behavioral paradigm and then sacrificed for neurogenesis study.

Mice were first randomized into 2 groups (N = 16 animals/group). The first group was treated with vehicle (0.9% NaCl plus 10% DMSO) and considered as control (non-stessed mice). The second group (CORT+) was given corticosterone (35 μg/ml/day, equivalent to 5 mg/Kg/day) available *ad libitum *in the drinking water in opaque bottles to protect it from light, for 7 weeks (stressed mice). After 4 weeks treatment mice were randomly assigned to four different conditions: CORT-/Vehicle (N = 8 mice); CORT-/*Hypericum perforatum *(N = 8 mice); CORT+/Vehicle (N = 8 mice); CORT+/*Hypericum perforatum *(N = 8 mice). *Hypericum perforatum *was prepared every day and dissolved in minimum volume of dimethyl sulfoxide (DMSO) plus saline solution (0.9% NaCl) with the final working concentration of 30 mg/Kg body weight. The final volume of DMSO in the *H. perforatum *vehicle was less than 10%. Daily intraperitoneal injections of *Hypericum perforatum *(30 mg/Kg body weight) or saline were performed for 3 weeks. The route of administration and dose were chosen in according to the study by Di Paola et al. [5]. Behavioral tests were performed before the treatments, after 4 weeks (CORT- and CORT+ groups) and 7 weeks (CORT-/Vehicle, CORT-/*Hypericum perforatum*, CORT+/Vehicle, CORT+/*Hypericum perforatum*). Mice were injected with BrdU (150 mg/Kg, i.p.) dissolved in saline and scarified after 2 hours (Figure 1).

### Fur state and body weight

Mice were inspected and weighted daily for a week before the CORT treatment, during the following 4 weeks of CORT treatment and during the *Hypericum perforatum *treatment. The condition of their fur was scored once a week by two independent observers blinded. Scores were averaged over the treatment periods for each animal. The coat-state evaluation involved the assessment of eight different body parts: head, neck, dorsal coat, ventral coat, tail, forepaws, hind paws and genital region. For each body area, a score of 0 was attributed for a coat in good condition or a score of 1 for a dirty coat. The total score was defined as the sum of the scores for each body part (Griebel 2002) [30].

### Behavioral assessments

#### Open field (OF)

Locomotor activity was monitored for 5 min in an open field, a white Plexiglas box 50 × 50 cm with its floor divided into 16 squares. Four squares were defined as the center and 12 squares along the walls as the periphery. Each mouse was gently placed in the center of the box and activity was scored as a line crossing when a mouse removed all four paws from one square and entered another. Before each trial, the chamber was cleaned with water containing a detergent. The animals' behaviour was videotaped. The line crossings and the time spent in the center were counted and scored.

#### Novelty suppressed feeding test (NSF)

The test was carried out during a 5 min period as previously described [31]. Briefly, the testing apparatus consisted of a plastic box (50 × 50 × 20 cm), the floor was covered with 2 cm of wooden bedding. Twenty-four hours before behavioral testing, animals were deprived of all food in the home cage. At the time of testing, a single pellet of food was placed on a white paper platform positioned in the center of the box. The test began started after the animal was placed in a corner of the box. The measure of interest was scored when the mouse was sitting on its haunches and biting with the use of forepaws. After this test, mice were transferred to their home cage and the amount of food consumed in 5 min was measured.

#### Forced swim test (FST)

The test is based on that described by Porsolt et al. [32]. A vertical glass cylinder (25 cm high, 14 cm in diameter) was filled with 27°C water to a depth of 20 cm. Each mouse was gently placed in the cylinder for 6 min and the duration of floating (i.e. the time during which mice made only the small movements necessary to keep their heads above water) was scored. Immobility time was analyzed during the last 4 min period of the test.

### Immunohistochemistry

After the brain was fixed for 1 week fixation at room temperature in buffered formaldehyde solution (10% wt/vol in PBS), samples were dehydrated in graded ethanol and embedded in Paraplast (Sherwood Medical, Mahwah, NJ). Thereafter, 7-μm sections were deparaffinized with xylene and rehydrated. BrdU and DCX analysis was carried out after boiling in 0.01 M citrate buffer for 4 min. Endogenous peroxidase was quenched with 0.3% (vol/vol) hydrogen peroxide in 60% (vol/vol) methanol for 30 min. Non-specific adsorption was minimized by incubating the section in 2% (vol/vol) normal goat serum in PBS for 20 min. Endogenous biotin or avidin binding sites were blocked by sequential incubation for 15 min with biotin and avidin (DBA, Milan, Italy), respectively. Sections were incubated overnight with 1) mouse monoclonal anti-BrdU antibody (1:100 in PBS, wt/vol) and with 2) goat polyclonal anti-Doublecortin antibody (Santa Cruz, California, USA ). Sections were washed with PBS and incubated with the secondary antibody. Specific labelling was detected with a biotin-conjugated goat anti-rabbit IgG and avidin-biotin peroxidase complex (DBA). The counterstain was carried out with nuclear fast red (red background). All sections were observed using light microscopy at 40X magnification (Axostar Plus equipped with Axio-Cam MRc, Zeiss) and studied via Imaging computer program (Axio-Vision, Zeiss). Quantitative evaluations was carried out on color monitor. For each experiment nine sections (three sections per mice) were selected for analysis. For each section,, the numbers of immunopositive cell for correspondinf antibodies were counted in dentate gyrus (DG).

### Golgi impregnation

FD Neurotech kit (FD NeuroTechnologies, Ellicott City, Md, USA) was used for Golgi impregnation of tissue. Blocks were placed directly into solutions A and B, without rinsing, and remained there for 2 weeks in the dark at room temperature. Forty-eight hours after placing in solution C (4°C), the blocks were frozen on dry ice and stored at -70°C until sectioning. Cryostat sections (100 μm) were cut at -25°C and mounted onto gelatinized slides. Slides were allowed to dry in the dark, and the rest of the staining process done as previously described [33]. Neurons were chosen for the analysis if completely impregnated with Golgi stain and unobscured by other impregnated neurons or precipitant. Moreover 70% of the dendritic tree was visible within the plane of focus and dentate granule neurons must be located in the outer one-half of the granule cell layer in DG. Cells chosen for analysis had to be well impregnated, clearly distinguishable from adjacent cells and have continuous unbroken dendrites. Spines were counted under oil (X100), using light microscopy (Axostar Plus equipped with Axio-Cam MRc, Zeiss), and the entire visible dendritic length measured by Imaging computer program (Axio-Vision, Zeiss). Spine density was calculated referring to the length of the dendrite.

### Statistical Analysis

All values in the figures and text are expressed as mean ± standard error of mean (S.E.M.) of N observations. For the *in vivo *studies N represents the number of animals studied. For all experiments two-way ANOVA was applied. Significant interactions were resolved using *post hoc *ANOVAs with adjusted *p *value.

## Results

### Behavioral mesurements

**Figure 2:**
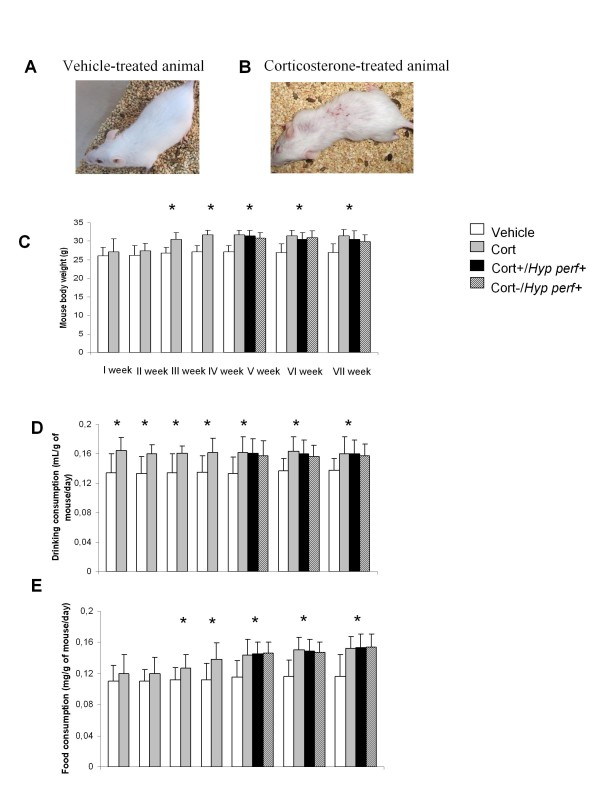
**Changes in the physiological parameters after corticosterone and *Hypericum perforatum *treatment**. (A,B) 4 weeks corticosterone treatment induced deterioration of the coat state (C) Mouse body weight of each animal was monitored during 4 weeks corticosterone (35 μg/ml/day) and *Hypericum perforatum *(30 mg/Kg) treatment (3 weeks). Values plotted are mean ± SEM (N = 8 per group). * p < 0.05 versus vehicle group. (D, E) Drinking and food consumption in each group of animal was observed during 4 weeks corticosterone treatment (35 μg/ml/day) and *Hypericum perforatum *(30 mg/Kg) treatment (3 weeks). Values plotted are mean ± SEM (N = 8 per group). * p < 0.05 versus vehicle group.

**Figure 3:**
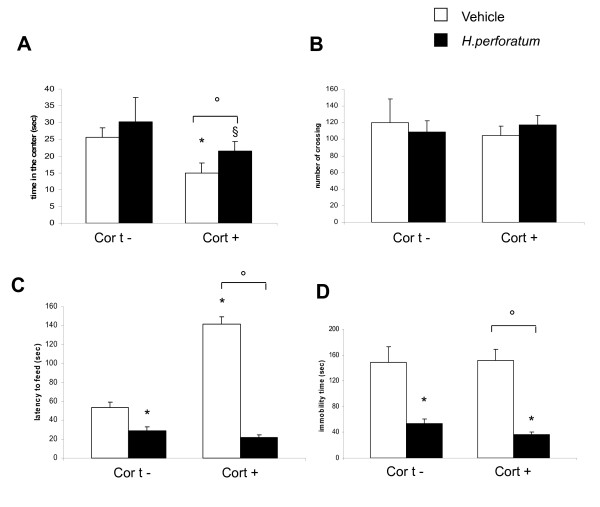
**Effect of chronic *Hypericum perforatum *treatment on corticosterone-induced behavioral changes**. (A) Effects of 3 weeks of *Hypericum perforatum *treatment, beginning after 4 weeks of corticosterone administration, on anxiety behaviors in OF. Anxiety is calculated as mean of the total time in the center in seconds. Exogenous chronic corticosterone treatment decreased the time spent in the center of arena. This anxiety phenotype was reversed by chronic administration of *Hypericum perforatum*. Values plotted are mean ± SEM (N = 8 per group). * p < 0.05, ° p < 0.05, § p < 0.05 versus vehicle group, corticosterone/vehicle group, and *Hypericum perforatum*/vehicle group, respectively. (B) Regarding the number of crossing, cort- and cort+ mice did not show differences after chronic corticosterone administration; exogenous *Hypericum perforatum *administration did not modify this parameter. Values plotted are mean ± SEM (N = 8 per group). (C) In the NSF paradigm, exogenous chronic corticosterone administration increased the latency to feed; this phenotype was reversed by chronic administration of *Hypericum perforatum*. Values plotted are mean ± SEM (N = 8 per group). * p < 0.05, ° p < 0.05, versus control group and corticosterone/vehicle group, respectively. (D) In the FST chronic corticosterone administration had no effect, while *Hypericum perforatum *treatment decreased the immobility time in both cort- and cort+ group. Values plotted are mean ± SEM (N = 8 per group). * p < 0.05, ° p < 0.05, versus control group and corticosterone/vehicle group, respectively.

Alterations in coat state were observed at the end of the corticosterone treatment in the presence or absence of 3 weeks *Hypericum perforatum*. Deterioration of the coat state, altered body weight, increased drinking and food consumption were provoked by a long term administration of exogenous corticosterone. These parameters were not significantly re-established under *Hypericum perforatum *treatment (30 mg/Kg/day) for 3 weeks (Figure 2). Exogenous administration of corticosterone developed an anxiety-like phenotype in the OF and NSF but not in FST. In particular, with regard to the OF, an evident decrease on the time spent in the center of arena was seen. Such alteration was then reversed by *Hypericum perforatum *treatment. (Figure 3A). The number of crossing in chronic corticosterone-treated mice did not show significant differences compared to the vehicle and remained unchanged after treatment with *Hypericum perforatum *(Figure 3B).

In the NSF test, exogenous chronic corticosterone administration increased the latency to feed while chronic administration of *Hypericum perforatum *reversed this anxiety phenotype (Figure 3C) without affecting the home food consumption.

Chronic corticosterone administration had no effect in the FST, whereas *Hypericum perforatum *administration decreased the immobility time in both corticosterone-treated and vehicle group (Figure 3D).

### Immunohistochemistry results

**Figure 4:**
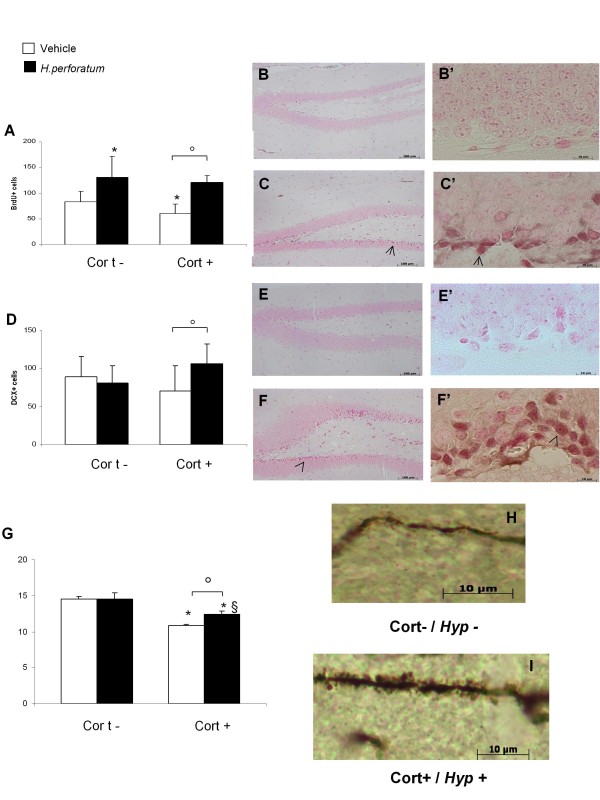
**Cell proliferation, dendritic maturation and synaptic plasticity in the dentate gyrus of the mice hippocampus**. (A) The number of BrdU+ cells decreased after exogenous corticosterone administration; this number significantly increased after *Hypericum perforatum *treatment. Values plotted are mean ± SEM (N = 8 per group). * p < 0.05, ° p < 0.05, versus control group and corticosterone/vehicle group, respectively. (B) BrdU of vehicle-treated groups at 40X magnification (B') BrdU of vehicle-treated groups at 100X magnification (C) BrdU of *Hypericum perforatum *at 40X magnification (C') BrdU of *Hypericum perforatum *at 100X magnification (D) The total number of DCX+ cells did not change after corticosterone administration. *Hypericum perforatum *treatment increased the number of DCX+ cells in corticosterone treated mice. Values plotted are mean ± SEM (N = 8 per group). ° p < 0.05, versus corticosterone/vehicle group. (E) DCX of vehicle-treated groups at 40X magnification (E') DCX of vehicle-treated groups at 100X magnification (F) DCX of *Hypericum perforatum *at 40X magnification (F') BrdU of *Hypericum perforatum *at 100X magnification (G) The number of spines in DG revealed a significant reduction in the development of dendritic spines in cort+ mice that was reverted by *Hypericum perforatum *treatment. Values plotted are mean ± SEM (N = 8 per group). * p < 0.05, ° p < 0.05, § p < 0.05 versus vehicle group, corticosterone/vehicle group, and *Hypericum perforatum*/vehicle group, respectively. (H) Photomicrograph illustrating the apical dendrites of granular cells in cort-/*Hyp- *group (I) Photomicrograph illustrating the apical dendrites of granular cells in cort+/*Hyp+ *group

Exogenous chronic corticosterone exposure mimicked the effect of chronic stress on cell proliferation, decreasing the number of both BrdU^+ ^and cells in the granule cell layer (GCL) being then restored by 3 weeks of *Hypericum perforatum *administration (Figure 4A). In corticosterone-treated mice tracing of the apical dendrites of DG granular cells revealed a significant reduction in the development of dendritic spines that was reverted by *Hypericum perforatum *treatment (Figure 4G).

## Discussion

In the present study we showed that chronic corticosterone treatment, similarly to what previously reported by David et al. [20], induces an affective phenotype which was reversed by chronic administration of the *Hypericum perforatum*. The pre-treatment with corticosterone leads to a deterioration of the state of the coat that can not be reversed by chronic *Hypericum perforatum *administration. For instance, an anxiogenic effect was observed in CORT+ mice in the OF and the NSF. This effect was reversed by *Hypericum perforatum *treatment. In addition, CORT+ mice showed deficit in hippocampal progenitor cell proliferation and a reduced number of CA3 dendritic spines which were prevented by *Hypericum perforatum *chronic treatment.

Consistent with previous findings [20], our behavioural data demonstrate that increased corticosterone levels induce anxiety in CD1 mice as assessed by the decreased time in the center of the arena in OF paradigm and by the increase in latency to feed in NSF. Corticosterone-treated mice did not show depression-like phenotype in the FST. *Hypericum perforatum *administration significantly ameliorated the anxiety-like phenotype (OF and NSF) in CD1 mice. Our data are in accordance with those reporting an anti-anxiety effect induced by *Hypericum perforatum *administration in models of restrain stress and sleep deprivation [34,35]. Studies conducted by Flausino et al. and Singewald et al. [36,37] have shown that chronic administration of *Hypericum perforatum *induced an antidepressant-like effect in Mg-depleted mice in the forced swim test, as well as anxiolytic-like effects in both anxiety tests. In our study, we used a different chronic stress paradigm and performed different behavioral paradigms (OF and NSF). Nonetheless, our data further reaffirm a role for *Hypericum perforatum *in helping to cope with different stressful conditions.

The synaptic correlates of this anti-anxiety effect are still a matter of debate. There is some preliminary evidence of the mode of action of *Hypericum extract *such as inhibition of uptake of serotonin (5-HT), noradrenaline, dopamine [8,38-42]. Based on the previous work hypericin was considered to be an inhibitor of both MAO type A and B [43] and inhibitor of synaptosomal reuptake of serotonin, noradrenaline and dopamine [7]. Furthermore, *Hypericum perforatum *extract increases extracellular levels of dopamine, noradrenaline, serotonin in the rat locus coeruleus [8] and modulated adenosine, GABA_A_, GABA_B _and glutamate receptors [39]. Nevertheless, whether these effects could account for the in-vivo anti-anxiety properties need still to be demonstrated.

To further verify the effects of *Hypericum perforatum *on hippocampal plasticity, we carried out an experiment on hippocampal progenitor cell proliferation. The hippocampus is a region that shows a remarkable capacity for structural reorganization. Preexisting neural circuits undergo modifications in dendritic complexity and synapse number, and entirely novel neural connections are formed through the process of neurogenesis. Stress exerts a significant modulation of hippocampal structural plasticity and is a potent negative regulator of neurogenesis. Different chronic stress suppress the rate of adult dentate gyrus proliferation and decreases the size of newborn cell clusters [45]. In accordance with these data, we found a reduction in hippocampal progenitor cell proliferation in mice chronically treated with corticosterone. The effects of corticosterone administration on neurogenesis are limitated to the proliferation stage and not the maturation of newborn neurons. Interestingly, the effect of *Hypericum perforatum *extract on proliferation and maturation is more pronounced in corticosterone-treated mice than in controls. We can speculate that this model may increase the dynamic range in which *Hypericum perforatum *extract exerts its effects on different stages of neurogenesis. Adult hippocampal neurogenesis in the adult hippocampus has been implicated in cognitive function and is stimulated by antidepressant drugs, although its functional impact and contribution to the aetiology of anxiety/depression is unclear [20,45,46]. However, severe or chronic stress exposure, both in early life as well as in adulthood, affect hippocampal neurogenesis and plasticity [46,47]. A reduction in neurogenesis can theoretically contribute to the cognitive symptoms of depression, even though by itself is unlikely to produce the full mood disorder [49]. The molecular mechanisms by which glucocorticoids induce these changes are however still unclear. Conversely, we demonstrated that in corticosterone-treated mice these changes are reverted by 3 weeks *Hypericum perforatum *administration.

*Hypericum perforatum *administration reverted the negative effect of stress on dendritic spine in mature hippocampal neurons. Dendritic spines are small specialized membranous protrusions that contain the postsynaptic machinery (PSD, glutamate receptors, cytoskeleton) and play a crucial role in synaptic plasticity [50] and calcium signaling [51-53]. Dynamic changes in spine number and morphology are closely linked to changes in strength of synaptic connections [54]. It has been suggested that a derangement of spine dynamics that favors loss of spines is a candidate mechanism for stress-evoked dendritic atrophy and associated synaptic dysfunction [55]. Our data confirm the negative effects of stress on dendritic spine number and demonstrated for the first time that *Hypericum perforatum *administration significantly decreased stress-induced dendritic pathology. Live imaging studies showed that spines are remarkably dynamic, changing size and shape and number over timescales of seconds to minutes [56]. Stress-induced changes in neurotransmitters, growth factors, hormones and oxidative stress could be responsible for the dendritic spines structural changes (size, number and shape). For instance, *Hypericum perforatum *administration significantly attenuated lipid peroxidation, nitrite concentration and partially restored GSH and catalase activity in chronic restrained mice suggesting a strong antioxidant effect [35]. In this respect, administration of fluoxetine also reduces oxidative stress in restraint animals [57] and promotes resilience. It is thus conceivable that antioxidant properties of *Hypericum perforatum *may be protective against the deleterious effects of chronic stress on hippocampul dendritic spines.

## Conclusion

This study establishes a previously unknown framework underlying both resilience and anti-anxiety action of *Hypericum perforatum*. Our results provide evidence of morphological adaptations occurring in both newborn and mature hippocampal neurons that might underlie resilient responses to chronic stress and that might contribute to the therapeutic effects of chronic *Hypericum perforatum *treatment.

## Competing interests

The authors declare that they have no competing interests.

## Authors' contributions

RC, ES, SC performed the study and drafted the manuscript. RC, AM carried out all behavioral tests in animals. EM participated in the design of the study and performed immunostaining. GLS, PB, FB helped with the statistical analysis and interpretation of the data. All authors read and approved the final manuscript.

## Pre-publication history

The pre-publication history for this paper can be accessed here:

http://www.biomedcentral.com/1472-6882/11/7/prepub
